# Relaxation Modes for Trapped Crystal Point Defects

**DOI:** 10.6028/jres.067A.030

**Published:** 1963-08-01

**Authors:** A. D. Franklin

## Abstract

Group representation theory is applied to the problem of calculating the relaxation modes of a point defect trapped near an impurity atom or other defect in a crystal, where more than one set of neighboring sites is available to the point defect. For illustration, the case of a cation vacancy trapped near a divalent impurity in the sodium chloride lattice is treated, including nearest- and next-nearest-neighbor sites.

Haven and van Santen [1]^1^ and Wachtman [2] have used group theory methods to determine the relaxation modes appropriate to a vacancy trapped in the vicinity of an impurity ion in an ionic crystal, Wachtman applied the results to a study of ThO_2_ containing Ca^++^ and oxygen ion vacancies. Haven and van Santen treated the case of a sodium-chloride lattice containing divalent impurities and sodium-ion vacancies. They gave complete solutions for all modes for the relaxation involving only nearest-neighbor positions, and also considered several special cases when next-nearest-neighbor positions were allowed as well. Previously, Lidiard [3] had solved, without restrictions, for the electrically active relaxation involving next-nearest-neighbor positions, but had not distinguished the two distinct modes present in his solutions. Dreyfus [4] showed that Lidiard’s solution was indeed equivalent to two electrically active modes. Haven and van Satnen had also obtained two electrically active relaxation modes.

The group theory methods can in fact be very readily extended to include any number of additional sets of neighbors. The problem of the undriven relaxation can be expressed mathematically in matrix form [2]:

$$\left(\frac{d}{dt}I+C\right)\;\vec{p}=0$$ (1)

where *I* is the identity matrix; *C* is a matrix in which the element in the *i*th row and *j*th column is the negative probability per unit time of a jump from the *j*th into the *i*th site, and in which the *i*th diagonal element is the sum of all jump probabilities out of the *i*th site; and $\vec{p}$ is the vector whose *i*th component is the probability of occupation of the *i*th site by the point-defect. The eigenvectors of *C* in the space of $\vec{p}$ are then the relaxation modes.

Since by definition the various sets are not carried into each other by the point-group operations of the crystal, they correspond to independent, orthogonal subspaces of the vector space defined by the occupation probability of the allowed sites. Hence the relaxation modes corresponding to the various appropriate irreducible representations of the point group can be found separately for each set, and then combined in a simple fashion. The relaxation modes for the whole system, involving all allowed sites, will be just linear combinations of the modes for the several sets, combining together only modes belonging to the same irreducible representation and arising, as partners, from the same symmetry-basis functions as discussed by Bethe [5].

Use of standard matrix algebra methods [6] allows the problem to be solved. A real, orthogonal transformation matrix may be formed from the uncombined relaxation modes as columns. This matrix transforms C to reduced form, from which the eigenvalues and the coefficients in the linear combinations of uncombined relaxation modes that constitute the eigenvectors may be calculated. The eigenvalues are just the reciprocal relaxation times. One eigenvalue is always zero, corresponding to the equilibrium distribution of the defects. If the corresponding normalized eigenvector is ${\vec{u}}_{1}$ and the other normalized eigenvectors are denoted by ${\vec{u}}_{i}$, then the solutions to eq (1) can be written

$$\begin{array}{l} \vec{p}=\left[{\vec{u}}_{1}+\sum_{i=2}^{N}f_{i}{\vec{u}}_{i}\right]{\left(\sum_{k}u_{1k}\right)}^{-1}\\ f_{i}=f_{i0}\exp[-\lambda_{i}t] \end{array}$$

where the *f_i_*_0_ are determined by the initial distribution, the *u*_1_*_k_* are the components of ${\vec{u}}_{1}$, and the λ*_i_* are the eigenvalues, and *N* is the total number of sites available.

Application of this technique to the problem of the motion of a sodium-ion vacancy trapped near a divalent impurity ion in the sodium-chloride lattice, and allowing jumps among nearest and next-nearest neighbors as well as interchange between the impurity ion and the vacancy on a nearest-neighbor site, results in the following (unnormalized) relaxation modes [7]:

**Table:** 

Mode number	Description	Irreducible representation [8]	Relaxation frequency	Occupation ratio, *a*
				
1	Equilibrium	*A*_1_*_g_*	0	*w*_3_*w*_4_
2	Hydrostatic	*A*_1_*_g_*	2(*w*_3_+2*w*_4_)	−2
3, 4, 5, 6	Uniaxial compression	*E*_2_*_g_*	$\left(3w_{1}+w_{3}+2w_{4}\right)\pm {\left[{\left(3w_{1}+w_{3}-2w_{4}\right)}^{2}+2w_{3}w_{4}\right]}^{\frac{1}{2}}$	$\left\{(3w_{1}+w_{3}-2w_{4})\mp {\left[{\left(3w_{1}+w_{3}-2w_{4}\right)}^{2}+2w_{3}w_{4}\right]}^{\frac{1}{2}}\right\}/w_{4}$
7, 8, 9	Shear	*T*_1_*_g_*	2(2*w*_1_+*w*_3_)	
10, 11, 12	Inactive	*T*_1_*_u_*	2(3*w*_1_+*w*_2_+*w*_3_)	
13, 14, 15, 16, 17, 18	Electrical	*T*_2_*_u_*	$(w_{1}+w_{2}+w_{3}+2w_{4})\pm {\left[{\left(w_{1}+w_{2}+w_{3}-2w_{4}\right)}^{2}+4w_{3}w_{4}\right]}^{\frac{1}{2}}$	$\left\{(w_{1}+w_{2}+w_{3}-2w_{4})\mp {\left[{\left(w_{1}+w_{2}+w_{3}-2w_{4}\right)}^{2}+4w_{3}w_{4}\right]}^{\frac{1}{2}}\right\}/w_{4}$

**Figure:**
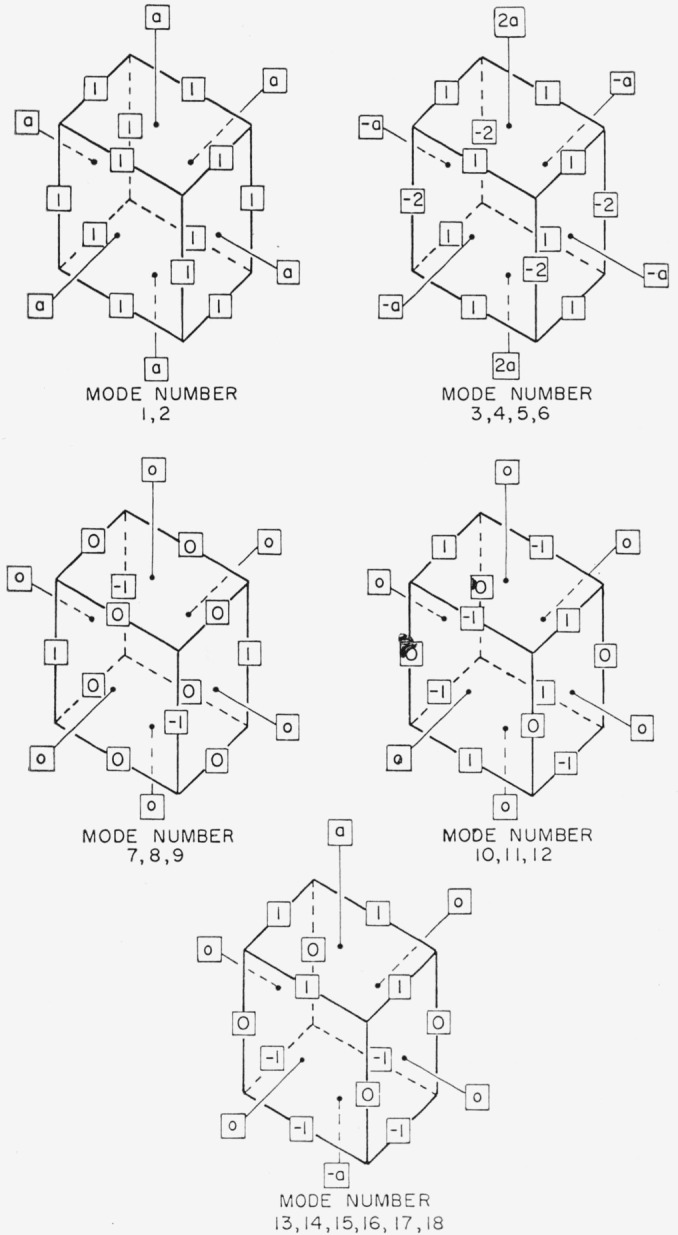


In this table, the *w*’s represent the jump frequencies for the unit motions, as follows: *w*_1_—between nearest-neighbor positions; *w*_2_—interchange of the impurity ion and the vacancy in a nearest-neighbor position; *w*_3_—nearest-neighbor to next-nearest-neighbor position; and *w*_4_—the reverse of *w*_3_. For any group, such as modes 7, 8, and 9, having the same relaxation frequency, any linear combination will also be a relaxation mode. In the group 3, 4, 5, and 6, there are two pairs distinguished by the cube axis along which the uniaxial compression acts. Note that relaxation along the third axis is the negative sum of those along the other two axes. In the electrically active group 13 to 18, there are three pairs, again corresponding to the three independent cube axes. The occupation ratios, *a*, given in the last column are defined in the sketches shown in the figure for the various modes.

## Footnotes

1 Figures in brackets indicate the literature references at the end of this paper.
